# α-Amylase in *Aspergillus oryzae*-fermented rice promotes the growth of human symbiotic *Faecalibacterium Prausnitzii*

**DOI:** 10.1038/s41598-026-36928-x

**Published:** 2026-01-20

**Authors:** Haruyuki Nakayama-Imaohji, Ayano Tada, Shuya Ogiwara, Emmanuel Munyeshyaka, Nafisa Tabassum, Tatsunori Mori, Rintaro Fujikawa, Keita Suzuki, Tomomi Kuwahara

**Affiliations:** 1 Department of Molecular Microbiology, Faculty of Medicine, Kagawa University, 1750-1 Ikenobe, Miki-cho, Kita-gun, 761-0793 Kagawa, Japan; 2 AuB Co. Ltd, 7-13-6 Ginza, Chuou-ku, Tokyo, 104-0061 Japan

**Keywords:** Amylase, Fermented rice, Aspergillus oryzae, Growth promotion, Faecalibacterium prausnitzii, Prebiotics, Biochemistry, Biotechnology, Microbiology

## Abstract

Diet is one of the critical factors that govern the human gut microbiome. Fermented foods provide beneficial effects for human health by supplying diverse nutrients and digestive enzymes. We aimed to investigate the effect of fermented rice extract prepared with *Aspergillus oryzae* on the growth of human symbiotic *Faecalibacterium prausnitzii*. After steamed rice was fermented by *A. oryzae*, a water-soluble fraction was prepared by centrifugation and used as rice-*koji* extract. The growth of *F. prausnitzii* in the media supplemented with 1% rice-*koji* extract was enhanced compared to that in a control medium. Fractionation of the rice-*koji* extract with an anion-exchange column and mass spectrometry analysis were conducted to identify the growth-promoting factors for *F. prausnitzii*. LC-MS/MS analysis revealed that the fractions showing a growth-promoting effect were rich in α-amylase from *A. oryzae*. The addition of purified *A. oryzae*-derived α-amylase and starch to the culture media increased the growth and butyrate production from *F. prausnitzii*. These findings suggest that *A. oryzae*-derived enzymes in fermented rice-*koji* possess a growth-promoting effect, enabling *F. prausnitzii* to efficiently uptake and utilize starch degradation products.

**Supplementary Information:**

The online version contains supplementary material available at 10.1038/s41598-026-36928-x.

## Introduction

 The human gut harbors a complex microbial ecosystem containing approximately thirty-eight trillion bacterial cells and is estimated to be composed of over 1,000 species^1^. Intensive investigation employing 16 S metagenomic analysis has demonstrated that gut microbiota closely associated with human health^2,3^. Gut microbiota provides health benefit to the host through nutritional and immunological ways^4–7^. As a result of fermentation of dietary fiber by specific gut microbes, short chain fatty acids (SCFA) represented by acetate, propionate and butyrate are produced in luminal contents. Gut microbiota-derived SCFA contributes to energy metabolism, maintenance of intestinal barrier function and differentiation of immune cells^8–11^. Among the SCFA, butyrate is giving rise to medical attention due to its critical role for tight junction integrity and induction of regulatory T cell in the intestine, suppressing the influx of microbial cell component into systemic circulation and excessive gut inflammation, respectively^8,12–15^. The reduction of butyrate-producing bacteria has been reported in various diseases such as obesity, type 2 diabetes, and inflammatory bowel diseases^15–20^. Based on these findings, butyrate-producing bacteria are considered as key stone gut microbes for host-microbe symbiosis^21,22^.

*Faecalibacterium* is one the butyrate-producing bacteria in human gut, which accounts for 5–15% of gut microbiota in healthy adults^23^. The abundance of *F. prausnitzii* has been reported to reduce in the metabolic disorders and inflammatory bowel diseases^24–29^. The bacterium shows health-promoting effect by anti-inflammation via specific protein^30^, extracellular polymeric matrix^31^ and/or butyrate production^32^, and increase energy metabolism through activation of mitochondria and upregulation of hepatic β-oxidation and lipid metabolism^33^. Therefore, *F. prausnitzii* is expected as a next-generation probiotics^27–36^. However, it seems to be difficult to produce commercially available probiotics employing *F. prausnitzii* because the bacterium is extremely oxygen-sensitive and inactivated within 20 min under aerobic condition^37^. From these backgrounds, many attempts have been made to investigate the prebiotic potential of dietary fiber for promoting the growth of indigenous *F. prausnitzii*. Inulin type fructan is widely used as prebiotics and enhances the growth *F. prausnitzii* as well as *Bifidobacterium*^38,39^. Laffin et al. reported that intake of amylose resistant starch increased the proportion of *Faecalibacterium* in endo-stage renal disease patients by randomized placebo-controlled study^40^. Recently, Tochio et al. revealed that 1-kestose (trisaccharide sugar consisting of a fructose residue of sucrose with fructose β-1,1-glycoside bond) showed superior prebiotic effect to other fructooligosaccharides with higher degree of polymerization of fructose, indicating that *F. prausnitzii* preferentially transports and utilizes the relatively short chain oligosaccharides^41,42^.

Fermented food using mold is one of the representative cultures in Asian countries and gathered attention to its health-promoting effects^43–45^. In Japan, *Aspergillus oryzae* is used for making “*koji*” by inoculating the mold on grains. *Koji* is utilized include sake (Japanese alcohol beverage), miso and various fermented foods. *Koji*-fermented food contained the diverse nutrients generated during fermentation process and digestive enzymes that facilitate the absorption of food components^46–48^. Recent studies have suggested that exogenous digestive enzymes in fermented foods, such as proteases and lipases, may function as prebiotics, beneficially influencing the composition of the gut microbiota and intestinal health^49^.

In this study, we investigated the prebiotic potential of *koji*-fermented rice on *F. prausnitzii.* The results of in vitro study showed that specific enzyme activity in *koji*-fermented rice facilitates the polysaccharide utilization of *F. prausnitzii* and promote the growth of this bacterium in the presence of starch as a sole carbon source.

## Results

### Rice-*koji* extract promoted *F. prausnitzii* growth

**Fig. 1:**
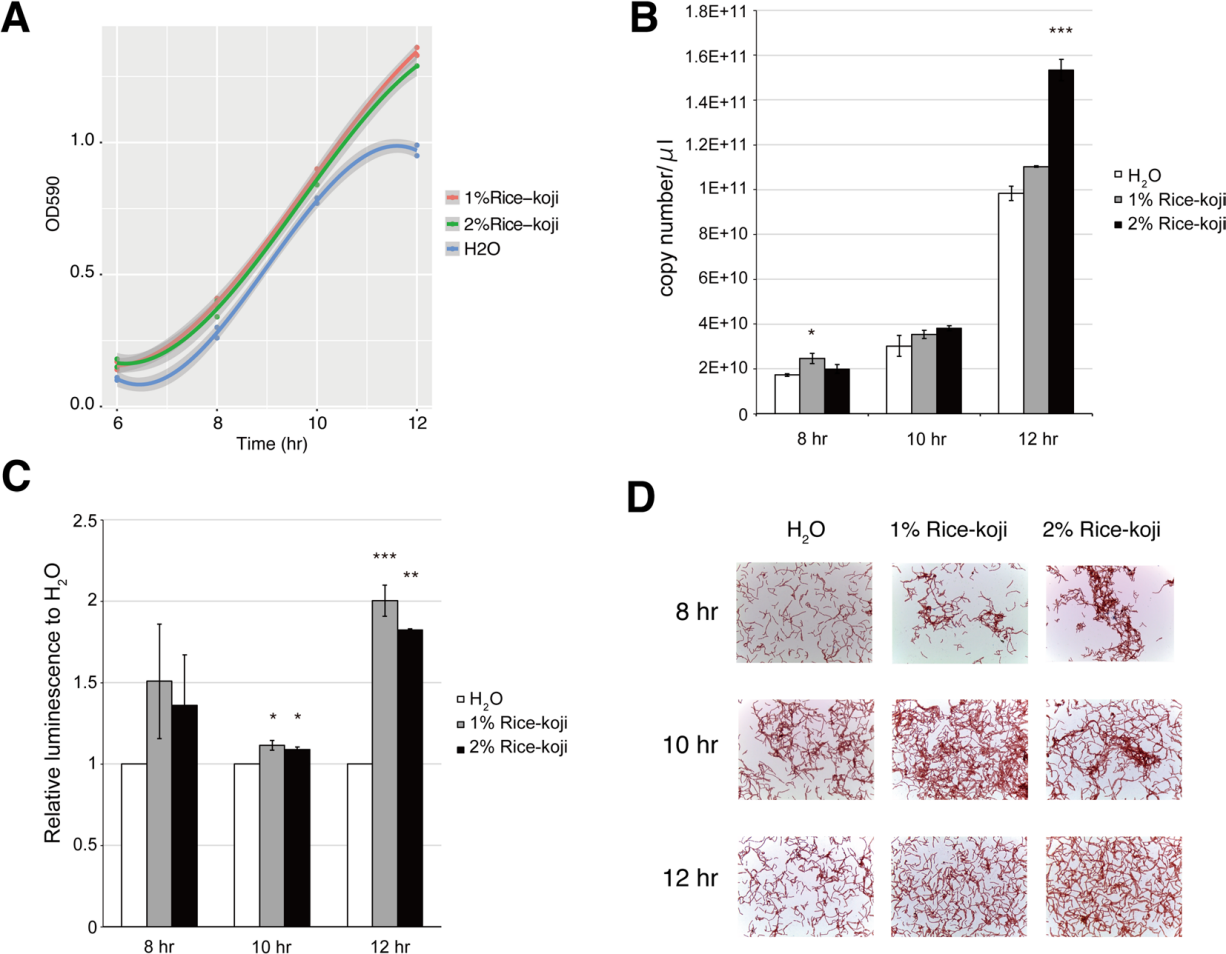
Effect of rice-*koji* extract on the growth of *F. prausnitzii*. **(A)** Growth monitoring by optical density at 590 nm. The control group (H₂O) received an equivalent volume of sterile distilled water to match the 1% or 2% extract concentrations. **(B)** Real-time PCR targeting the 16 S ribosomal RNA gene to compare the cell multiplication. Statistical significance was determined by one-way ANOVA with Dunnett’s *post-hoc* test against the H_2_O control (*, *p* < 0.05; ***, *p* < 0.001). **(C)** ATP production level in the culture media. Statistical significance was determined by one-way ANOVA with Dunnett’s *post-hoc* test against the H_2_O control (*, *p* < 0.05; **, *p* < 0.01; ***, *p* < 0.001). **(D)** Gram-staining of *F. prausnitzii* in the media with or without rice-*koji* extract.

Rice-*koji* extract was added to *Faecalibacterium* medium (FM) to the final concentration of 1.0% (v/v) or 2.0% (v/v). The growth of *F. prausnitzii* in these media was compared with that in FM added with distilled water in place of rice-*koji* extract (Fig. 1). Supplement with rice-*koji* extract at either dose enhanced the growth and optical density at 590 nm after 12-h cultivation was reached over 1.2, while the maximum growth of *F. prausnitzii* in control medium was 1.0, and the growth entered decline phase around 12 h after incubation (Fig. 1A). The genomic copy of cultured *F. prausnitzii* was determined by real-time PCR (Fig. 1B). The genomic copy number was increased by addition of rice-*koji* extract with dose-dependent manner. Correspondingly, ATP production in *F. prausnitzii* after 12-h incubation was significantly elevated in response to rice-*koji* extract (Fig. 1C). Periodical Gram-staining of the culture confirmed the growth promoting effect of rice-*koji* extract on *F. prausnitzii* (Fig. 1D). Cell density in FM supplemented with rice-*koji* extract was higher than in water control after 10-h cultivation. *F. prausnitzii* cells in FM supplemented with rice-*koji* extract tended to be aggregative.

### Identification of growth-promoting factors for *F. prausnitzii* in rice-*koji* extract

**Fig. 2:**
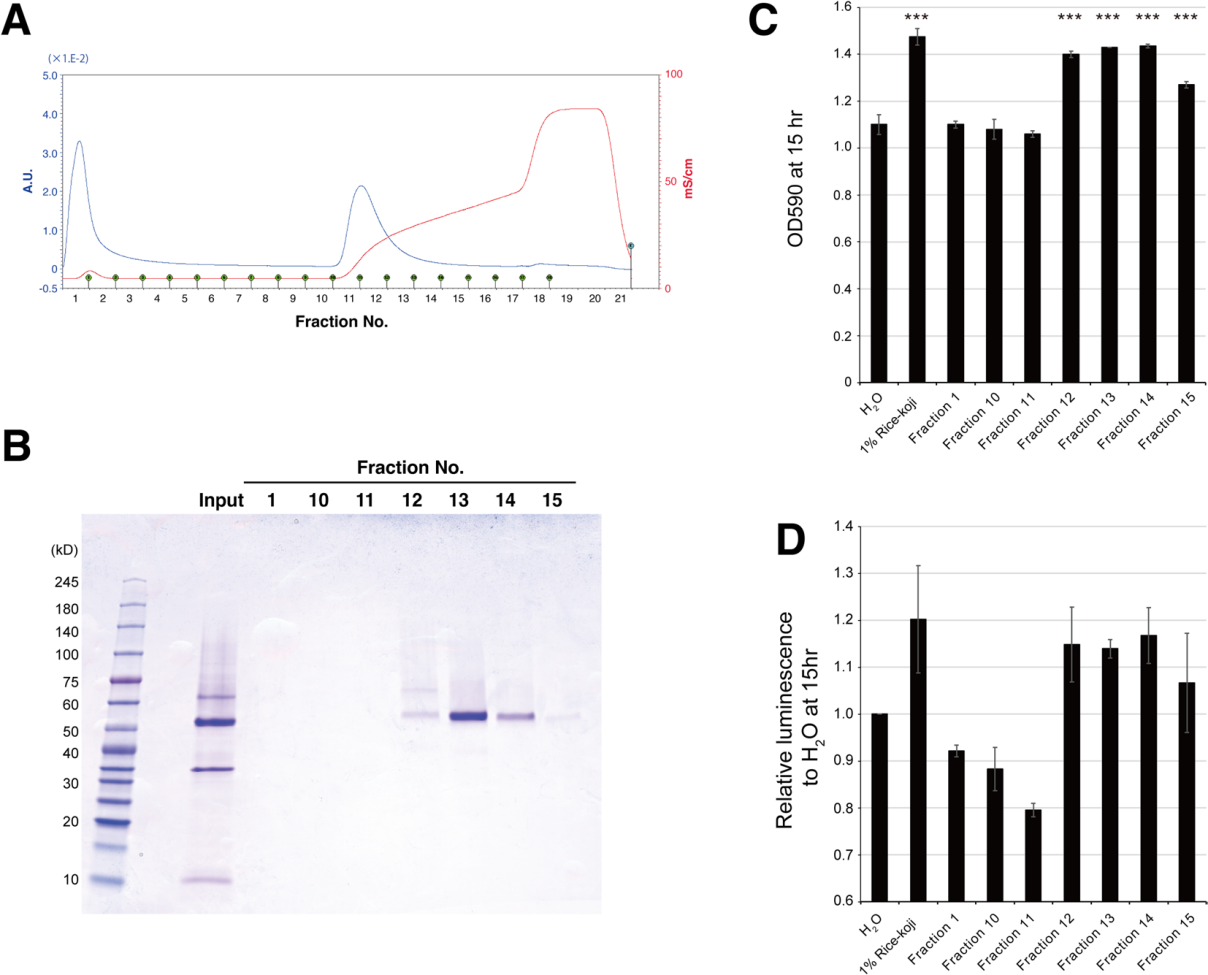
Fractionation of rice-*koji* extract by anion exchange chromatography **(A)** Anion exchange chromatography with UNOsphere Q column. After 90% ammonium sulfate precipitation of rice-*koji* extract was conducted, the sample was applied to UNOsphere Q column. Elution was performed with continuous gradient of 50 mM Tris-HCl, 1 M NaCl (pH7.5) and divided into 20 fractions. **(B)** SDS-PAGE of the original sample and indicated fractions. **(C)** Growth promoting effect of each fraction on *F. prausnitzii*. Optical density of the *F. prausnitzii* culture in the media supplemented with indicated fraction to 1% final concentration (v/v). Data were obtained from 15-h culture. Statistical significance was determined by one-way ANOVA with Dunnett’s *post-hoc* test against the H_2_O control (***, *p* < 0.001). **(D)** ATP production of *F. prausnitzii* after 15-h cultivation in the media supplemented with indicated fraction to 1% final concentration (v/v). No significant differences from the H_2_O control group were detected by Dunnett’s test.

Since the growth-promoting effect was heat-labile, suggesting a proteinaceous nature, the rice-*koji* extract was precipitated with ammonium sulfate and then fractionated using an anion exchange column to identify the growth-promoting substances for *F. prausnitzii* in the extract. We obtained a total 20 fractions. Elution peak was observed around fraction 11 to 12 (Fig. 2A). Correspondingly, SDS polyacrylamide electrophoresis detected proteinaceous band around 55 kDa from fraction 12 to 15 with highest densities observed in fraction 13 (Fig. 2B). We next examined the effect of each fraction on *F. prausnitzii* growth. As shown in Fig. 2C, the growth-promoting activity were detected in fraction 12 to 15, of which effect was slightly lower compared with original rice-*koji* extract. Correspondingly, ATP production in *F. prausnitzii* was enhanced in response to same fractions (Fig. 2D).

### LC-MS/MS analysis of fraction 13

To identify the growth-promoting molecules in rice-*koji* fraction, tandem liquid chromatography-mass spectrometry (LC-MS/MS) was conducted with the fraction 13. LC-MS/MS identified 22 proteins in this fraction. As shown in Table 1, the abundant molecule in fraction 13 was α-amylase of *A. oryzae* origin, of which molecular size was consistent with the band detected by SDS-PAGE (Fig. 2B). Peptidases were also detected as major components in this fraction.

**Table 1:**
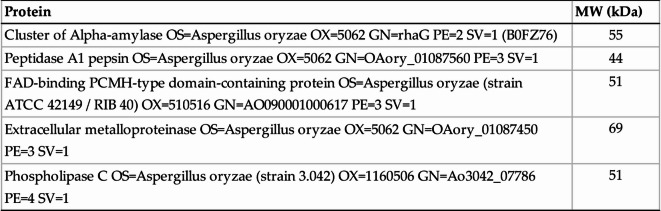
Top 5 list of the identified proteins in fraction 13 by LC/MS analysis.

### α-Amylase activity in the rice-*koji* fraction sample

**Fig. 3:**
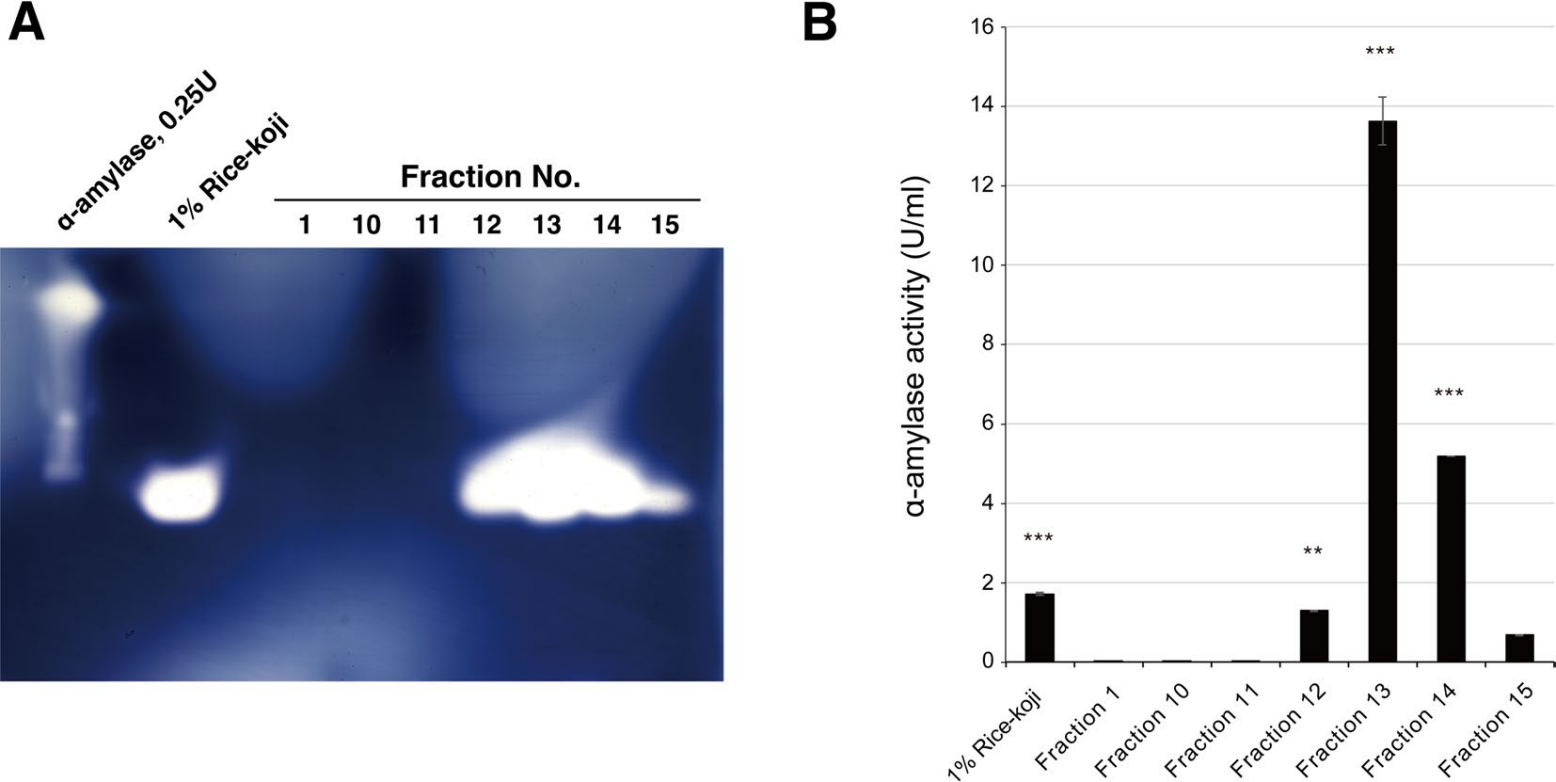
Detection of α-amylase activity in the rice-*koji* fraction. **(A)** Zymography of starch-containing acrylamide gel. SDS-PAGE of control α-amylase, rice-*koji* extract and the indicated fractions was conducted. The starch in the gel was stained with iodine solution. Original SDS-PAGE gel images are presented in Supplementary Fig. S3. **(B)** Measurement of α-amylase activity in the indicated rice-*koji* fractions. Note: Fraction 13 was concentrated during the purification process (see Methods), resulting in higher enzymatic activity per unit volume compared to the original rice-*koji* extract. Statistical significance was determined by one-way ANOVA with Dunnett’s *post-hoc* test against the Fraction 1 control (**, *p* < 0.01; ***, *p* < 0.001).

We determined whether rice-*koji* fractions (fractions 12–15), which promoted *F. prausnitzii* growth, contained α-amylase. The zymography using acrylamide gel containing starch clearly demonstrated that α-amylase activity was present in fractions 12–15 with fraction 13 showing the highest activity (Fig. 3A). Correspondingly, the highest α-amylase activity was observed in fraction 13, and most of the enzyme was condensed in this fraction when compared with original rice-*koji* (Fig. 3B). The other fractions did not contain α-amylase activity. To confirm that this growth-promoting effect was attributable to enzymatic activity, we prepared a heat-inactivated rice-*koji* extract. The α-amylase activity in the extract was completely abolished by the heat treatment (100 °C for 10 min) (see Supplementary Fig. S1A online). When this heat-inactivated extract was added to the culture, the growth-promoting effect on *F. prausnitzii* was completely lost, with the OD590 value being comparable to that of the water control (see Supplementary Fig. S1**B** online). These results demonstrate that the growth-promoting factor in the rice-*koji* extract is heat-labile and its effect is dependent on its catalytic activity.

### Growth-promoting effect of α-amylase-rich rice-*koji* fraction on *F. prausnitzii*

**Fig. 4:**
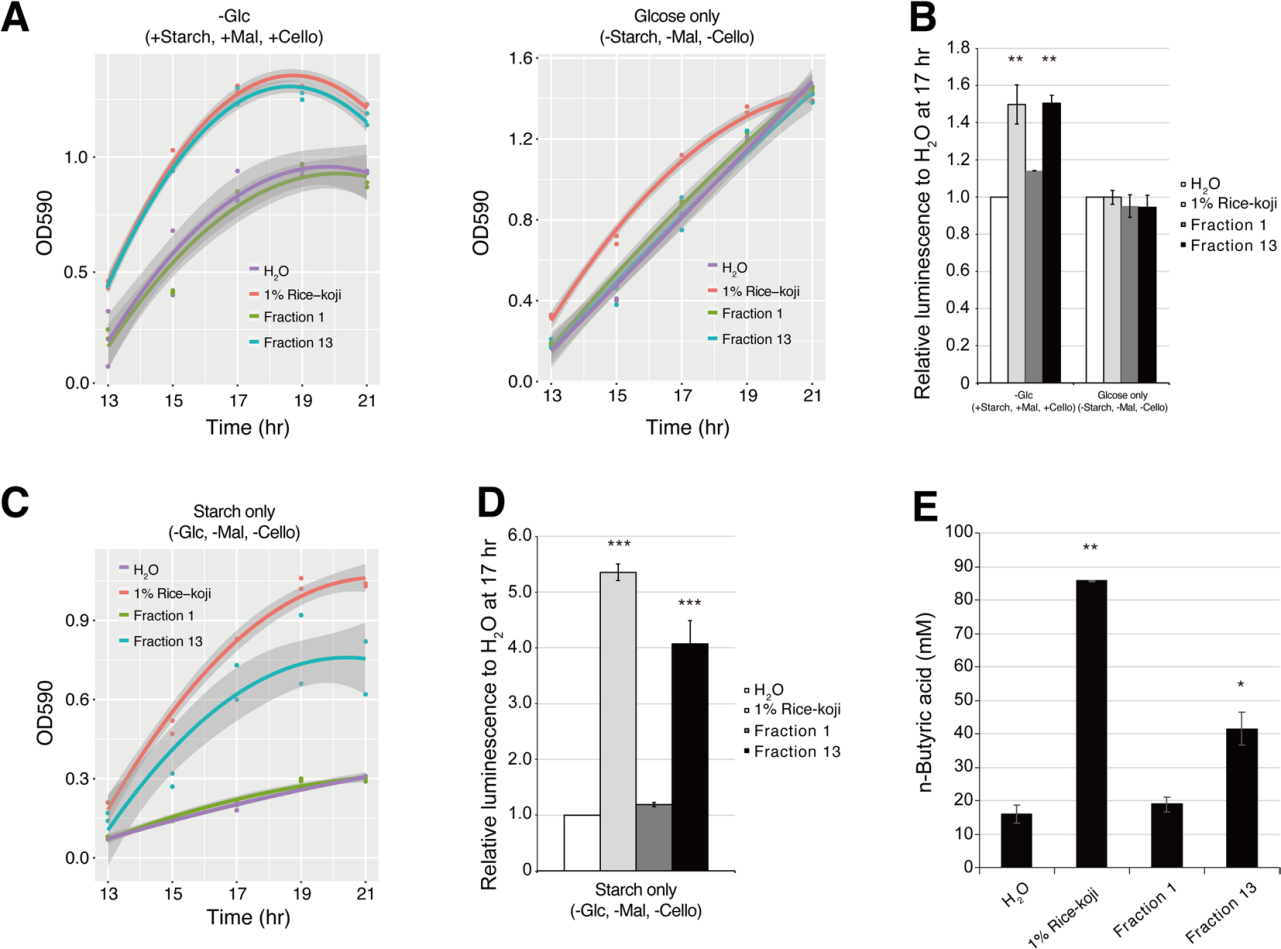
Effect of α-amylase-rich rice-*koji* fraction on *F. prausnitzii* growth. **(A)** Comparisons of growth enhancement effect of α-amylase-rich (fraction 13) and α-amylase-less (fraction 1) fractions on *F. prausnitzii*. The effect was evaluated in the presence or absence of D-glucose. The latter was supplemented with starch, maltose and cellobiose for carbon source. Growth curves are shown for H₂O (purple line), 1% Rice-*koji* (red line), Fraction 1 (green line), and Fraction 13 (blue line). **(B)** ATP production of *F. prausnitzii* after 17-h cultivation in the media described in panel A. Bars represent H₂O (white), 1% Rice-*koji* (light gray), Fraction 1 (dark gray), and Fraction 13 (black). Statistical significance was determined by one-way ANOVA with Dunnett’s *post-hoc* test against the H_2_O control (**, *p* < 0.01). **(C)** Growth promoting effect of α-amylase-rich (fraction 13) and α-amylase-less (fraction 1) fractions on *F. prausnitzii*. The effect was evaluated in the medium containing only starch as a carbon source. Growth curves are shown for H₂O (purple line), 1% Rice-*koji* (red line), Fraction 1 (green line), and Fraction 13 (blue line). **(D)** ATP production of *F. prausnitzii* after 17-h cultivation in the media described in panel C. Bars represent H₂O (white), 1% Rice-*koji* (light gray), Fraction 1 (dark gray), and Fraction 13 (black). Statistical significance was determined by one-way ANOVA with Dunnett’s *post-hoc* test against the H_2_O control (***, *p* < 0.001). **(E)** *n*-Butyric acid production by *F. prausnitzii*. The analysis was performed on culture supernatants harvested after 17 h of cultivation in a modified FM containing 0.1% starch as the sole carbon source, supplemented with 1% rice-*koji*, α-amylase-less (fraction 1) or α-amylase-rich (fraction 13) fraction. Statistical significance was determined by one-way ANOVA with Dunnett’s *post-hoc* test against the H₂O control (*, *p* < 0.05; **, *p* < 0.01).

We aimed to determine whether α-amylase promotes the growth of *F. prausnitzii*. Three kinds of media were prepared: the first contained D-glucose polymer (starch, maltose and cellobiose) but without D-glucose, the second and third contained monosaccharide D-glucose or starch as a sole carbon source, respectively. As shown in Fig. 4A, fraction 13 enhanced the growth of *F. prausnitzii* at the equivalent degree with original rice-*koji* in the presence of starch, maltose and cellobiose as carbon sources. On the other hand, no growth promotion was detected in fraction 1 that contained no α-amylase activity. The growth promoting effect of fraction 13 was not observed when *F. prausnitzii* was cultured in the medium containing D-glucose as a sole carbon source. However, original rice-*koji* showed growth promotion at mid-logarithmic phase. Correspondingly, original rice-*koji* and fraction 13 elevated ATP production only when *F. prausnitzii* was cultured in the media supplemented with D-glucose polymer, and the effect was not observed in the medium containing D-glucose as a sole carbon source (Fig. 4B). In the case of the media containing starch as a sole carbon source, growth-promoting effect of fraction 13 was observed although the effect was lower than original rice-*koji* (Fig. 4C). Consistent with the results, ATP production in this medium was elevated by the addition of fraction 13, of which effect was lower than original rice-*koji* (Fig. 4D). SCFA analysis was conducted to determine whether rice-*koji* extract increases butyrate production in *F. prausnitzii*. Rice-*koji* extract increased the butyrate production in the in vitro culture supplemented with starch (Fig. 4E). Addition of fraction 13 containing α-amylase also promoted the butyrate production in *F. prausnitzii*. These results indicate that *A. oryzae*-derived α-amylase in rice-*koji* extract facilitates the utilization of starch by *F. prausnitzii*.

### Effect of *A. oryzae*-derived α-amylase on *F. prausnitzii* growth

**Fig. 5:**
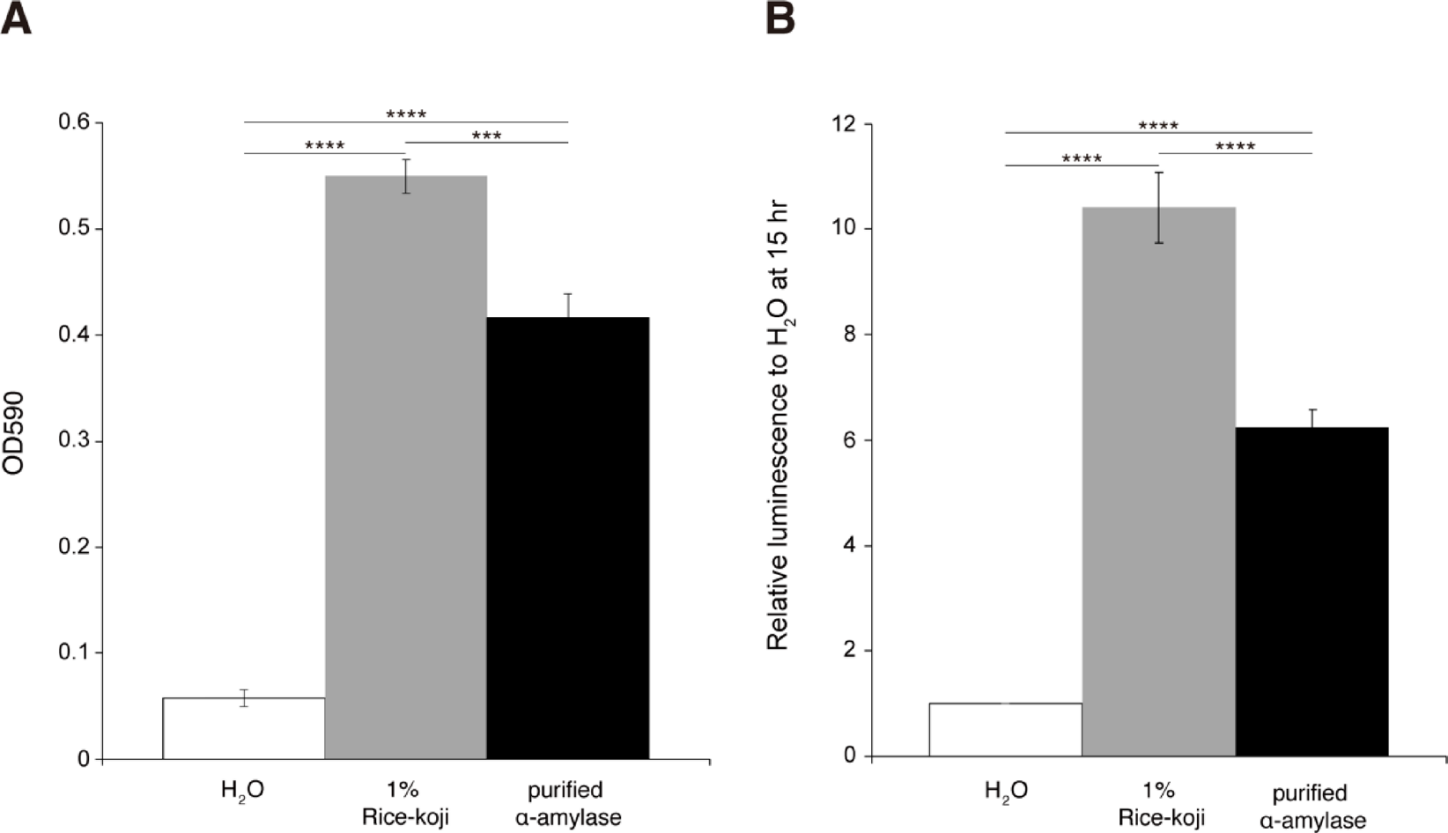
Effect of *A. oryzae*-derived α-amylase on the growth of *F. prausnitzii*. *F. prausnitzii* was cultured for 15 h in modified FM containing 0.1% starch as a sole carbon source, supplemented with either 1% rice-*koji* extract, an equivalent amount (56.4 mU) of purified α-amylase, or H₂O (control). **(A)** Growth promoting effect of α-amylase on *F. prausnitzii*. Bars represent H₂O (white), 1% Rice-*koji* (light gray), and 56.4 mU purified α-amylase (black). Statistical significance was determined by one-way ANOVA with Tukey’s *post-hoc* test (***, *p* < 0.001; ****, *p* < 0.0001). **(B)** ATP production of *F. prausnitzii* after cultivation in the respective media. Bars represent H₂O (white), 1% Rice-*koji* (light gray), and 56.4 mU purified α-amylase (black). Statistical significance was determined by one-way ANOVA with Tukey’s *post-hoc* test (****, *p* < 0.0001).

To determine whether *A. oryzae*-derived α-amylase contained in rice-*koji* extract contributes to the growth promotion of *F. prausnitzii*, we finally compared the cell density and ATP level after 15-h cultivation in modified FM containing 0.1% starch as a sole carbon source. The experiment compared cultures supplemented with either 1% rice-*koji* extract or an equivalent amount of purified *A. oryzae*-derived α-amylase (56.4 mU), against an unsupplemented control (H₂O). As shown in Fig. 5, the purified *A. oryzae*-derived α-amylase significantly enhanced growth (Fig. 5A) and ATP production (Fig. 5B) compared with the control (H₂O), but its effect was significantly lower than that of the rice-*koji* extract. These results indicate that while the high α-amylase content of the rice-*koji* extract contributes to its effect, its performance cannot be explained by the enzyme quantity alone and suggests the presence of other synergistic factors.

## Discussion

In this study, we identified that α-amylase derived from the *koji* mold is one of the primary components contributing to the growth-promoting effect of rice-*koji* water extract on *F. prausnitzii*. These findings indicate a novel functionality of fermented foods by *koji* mold and highlight important implications for developing new strategies to improve the gut environment by targeting *F. prausnitzii*, a promising next-generation probiotic.

Prebiotics are generally defined as a substrate that is selectively utilized by host microorganisms conferring a health benefit, and many of them are non-digestible carbohydrates^50,51^. Regarding *F. prausnitzii*, it is widely known that dietary fibers such as inulin, fructooligosaccharides (FOS), and resistant starch act as prebiotics^34,35^. From these studies, it is suggested that *F. prausnitzii* possesses its own endogenous enzymes, such as β-fructosidase and a variety of carbohydrate-active enzymes, to break down and assimilate these complex polysaccharides^34,35^. However, our results indicate that an exogenous digestive enzyme derived from the food matrix itself can function as a potent growth-promoting factor. It is thought that the *koji*-derived α-amylase is not directly utilized as a nutrient source by *F. prausnitzii*, but instead acts as a catalyst, breaking down starch present in the medium into more easily assimilable oligosaccharides (e.g., maltose, maltotriose, and dextrins). Furthermore, one of the key findings in this study is that the crude extract of rice-*koji* showed a more pronounced growth-promoting effect compared to the purified α-amylase. This effect may be attributable not only to its high amylase content but also to the synergistic effects of a diverse group of enzymes. *A. oryzae* is known to produce not only α-amylase but also glucoamylases and debranching enzymes. It is presumed that this enzyme cocktail works in concert to more efficiently break down starch into a mixture of oligosaccharides that are more easily fermented by *F. prausnitzii*. Consequently, it is suggested that rice-*koji* extract is a potential source of not only prebiotic oligosaccharides generated during the fermentation process, but also functional enzymes that facilitate the efficient utilization of polysaccharide substrates by *F. prausnitzii.* These findings are consistent with existing knowledge regarding the metabolic preferences of *F. prausnitzii*. Although *F. prausnitzii* can ferment a variety of substrates, it has been suggested to prefer relatively low-molecular-weight carbohydrates. A study by Tachio et al. reported that the trisaccharide 1-kestose (GF2) exhibited a stronger prebiotic effect on *F. prausnitzii* than the longer-chain FOS component, nystose (GF3)^41^. This suggests that the initial depolymerization of large polysaccharides may be a rate-limiting step in the metabolism of *F. prausnitzii*.

Every metabolic process comes with an energy cost. For bacteria, synthesizing and secreting enzymes to break down large extracellular polymers require a significant investment of ATP and cellular resources^52^. Although *F. prausnitzii* possesses the necessary enzymes for the depolymerization of complex polysaccharides, their efficiency may not be optimal for rapid growth compared to the utilization of pre-digested, smaller oligosaccharides. Therefore, it is speculated that the rice-*koji* extract, by providing digestive enzymes in the environment, allows *F. prausnitzii* to bypass this initial energy investment.

Another intriguing finding is that only the crude extract, and not the α-amylase-rich fraction 13, promoted growth in the D-glucose-only medium (Fig. 4A). This strongly suggests that the rice-*koji* extract contains a growth-promoting factor that is independent of the polysaccharide degradation mechanism. These factors could be micronutrients such as peptides, amino acids, and vitamins that were either released from the rice during fermentation or derived from the *koji* mold itself. This highlights the multifaceted potential of *koji*-fermented foods to support beneficial gut bacteria not only through enzymatic assistance but also through direct nutritional supply. The identification of these amylase-independent factors is an important research topic for the future.

The health-promoting effects of traditional Japanese foods made with *koji*, such as amazake, miso, and shio-*koji*, are widely recognized. These benefits have historically been attributed to the nutritional profile of the final product, such as improved bioavailability of nutrients, production of vitamins and essential amino acids, and the presence of prebiotic oligosaccharides^43,53^. However, our present findings suggest that enzymatic support for key commensal bacteria, such as *F. prausnitzii*, may also contribute to these effects. This concept of enzymatic support, or enzyme supplementation, can be considered a potential new design strategy for synbiotics and functional foods^49^. Since α-amylase production in *A. oryzae* is induced by maltose and starch^54,55^, rice-*koji* itself can be regarded as a good example of a synbiotic that provides both prebiotic polysaccharides (starch) and an assistant enzyme (α-amylase) for *F. prausnitzii*.

The limitation of this study is its in vitro model using a monoculture of *F. prausnitzii*. The human colon is a complex and competitive ecosystem where hundreds of microbial species coexist. In such an environment, the oligosaccharides produced from starch by α-amylase would competitively consume not only by *F. prausnitzii* but also by many other saccharolytic bacteria. The outcome of such competition cannot be predicted from our simple model. Furthermore, the substrate concentration and enzyme amount used in our in vitro model were determined based on experimental detection sensitivity and do not necessarily reflect the complex and fluctuating nutritional environment of the human gut. Moreover, before applying these findings to animal models, it will be crucial to investigate the optimal dosages of both the starch substrate and the α-amylase enzyme required to effectively support the growth of *F. prausnitzii* in a competitive in vivo environment. In addition to environmental and dosage factors, the physiological stability of the enzymes during gastrointestinal passage acts as another critical determinant for in vivo efficacy. Fungal enzymes, including α-amylase, are generally susceptible to degradation by gastric acid and host digestive proteases. Therefore, it remains to be determined whether these enzymes can retain sufficient catalytic activity upon reaching the colon or if protective strategies, such as encapsulation, are required to ensure their delivery. However, due to the current lack of a stable mouse model for mono-colonization with *F. prausnitzii*, human clinical intervention trials should also be conducted. In addition, genus *Faecalibacterium* is now understood to consist of multiple species and different phylogenetic groups^56^. It is crucial to investigate whether the growth-promoting effect of *koji*-derived α-amylase is a universal trait across the genus or specific to certain strains or clades with different carbohydrate utilization preferences.

## Methods

### Bacterial strains and isolation methods

The *F. prausnitzii* strain used in this study was isolated from a healthy male (marathon athlete) after obtaining written informed consent. This study was conducted according to the declaration of Helsinki and ethical guidelines for medical and biological research involving human subjects published from Ministry of Education, Culture, Sports, Science, Ministry of Health, Labour and Welfare and Ministry of Economy, Trade and Industry Technology in Japan. The experimental protocol was approved by Ethics Committees in Kagawa University (Approved No: H30-066). The stool specimen was quickly placed in an AnaeroPouch system (Mitsubishi Gas Chemical Co. Ltd., Japan) and stored under anaerobic conditions. All subsequent isolation work was performed in an anaerobic box (80% N_2_, 10% CO_2_, and 10% H_2_). One gram of stool specimen was collected and suspended in 10 ml of anaerobic diluent (4.5 g/L KH_2_PO_4_, 6.0 g/L Na_2_HPO_4_, 0.5 g/L L-cystein·HCl·H_2_O, 0.5 g/L tween 80, 1.0 g/L agar), then homogenized and diluted serially 10 to 10^8^-fold with the anaerobic diluent. The serial dilutions were plated onto modified ATCC medium1703 medium (see below) and incubated at 37°C for 2 days. The resulting colonies were subjected to PCR screening using *Faecalibacterium*-specific primers (Faepz-F1: 5’-GGGAGCGCAGGCGGGAAG-3’; Faepz-R1: 5’-TCAGCGTCAGTTGGTGCCCAGTAG-3’). Positive colonies were finally identified by 16 S rRNA gene sequencing.

## Growth medium

Modified ATCC medium1703 medium consists of (per liter of distilled water) 30 g of Trypticase peptone (BD); 5 g of yeast extract; 20 g of Lab-Lemco Powder (Oxoid); 5 g of K_2_HPO_4_; 1 mg of resazurin; 0.5 g of L-cysteine·HCl·H_2_O; 1 mg of vitamin K1; 5 µg of hemin; 15% filtered rumen fluid; 1 g of starch; 4 g of glucose; 1 g of cellobiose; and 1 g of maltose. This medium was designated as *Faecalibacterium* medium (FM) in this study. The medium was autoclaved at 115 °C for 15 min after adjusting the pH to 7.0. Carbohydrates were filter sterilized and added after autoclaving. Both liquid and agar media were prepared in anaerobic box immediately after sterilization.

### Preparation of rice-*koji*

200 g of milled Nanatsuboshi rice from Hokkaido, which was purchased from a market in Takamatsu city, Kagawa, Japan, was mixed with 300 ml of distilled water in a 500 ml beaker and allowed the rice to soak for about 1 day. The rice was placed in a colander and drained for about 1 h. Rice was steamed for about 40 min using an electric rice steamer (EM-185, MK SEIKO CO., LTD., Japan). The steamed rice was spread on a piece of Saran wrap and allowed to cool. In a sterilized 500 ml beaker, added 50 g of cooled steamed rice, 0.2 g of seed *koji* (BF-1, Higuchi Matsunosuke Shoten Co., Ltd., Japan), and 70 ml of sterilized distilled water, mixed evenly, and fermented at 30 °C for 3 days. The fermented *koji* solution was squeezed into a 50 ml centrifuge tube, centrifuged at 10,000 × *g* for 10 min, and the supernatant was collected. The resulting *koji* solution was sterilized through a 0.22 μm-filter and designated as rice-*koji* extract.

### Growth monitoring of *F. prausnitzii* strains in rice-*koji*-containing broth

The growth of *F. prausnitzii* was evaluated by absorbance at 590 nm, determination of ATP produced during growth, and quantitative PCR using primers Faepz-F1 and Faepz-R1. The growth of *F. prausnitzii* in FM broth with or without rice-*koji* (1 or 2%) or purified fractions of rice-*koji* extract (1%) was monitored by measuring OD590 after the overnight culture was inoculated into the fresh media (1.0% v/v). In the control group, an equivalent volume of sterile distilled water was added to the medium in place of the rice-*koji* extract to match the final volume. The culture supernatant and bacterial cells were collected at each measurement point. The amount of ATP in the culture supernatant was evaluated by measuring luciferase activity using the BacTiter-Glo Microbial Cell Viability Assay Kit (Promega). For quantitative PCR, genomic DNA was extracted from the collected cells using the Easy-DNA purification kit (ThermoFisher scientific), and subsequently amplified using TB Green Premix Ex Taq II (Takara) under the following conditions: preheating at 95 °C for 30 s and 40 cycles of 95 °C for 5 s, 60 °C for 30 s and 72 °C for 30 s in a Thermal Cycler Dice Real Time System III (Takara). All samples were run in triplicate. The copy number of 16 S rRNA gene was calculated from a standard curve using genomic DNA.

### Purification of growth-promoting substances in the rice-*koji* extracts

Ammonium sulfate at 90% saturated concentration was added to the rice-*koji* solution and completely dissolved for 2 h at 4 °C while stirring with a stirrer. The protein was salted out by centrifugation at 2000 × g for 30 min. Precipitated proteins were dissolved in 50 mM Tris-HCl pH 7.5 and dialyzed in the same buffer. The 90% ammonium sulfate precipitated fraction (10 mg) was applied to a UNOsphere Q anion column (BIO-RAD) on BioLogic LP system (BIO-RAD). The elution was performed by a linear gradient of 50 mM Tris-HCl (pH 7.5) and 50 mM Tris-HCl containing 1 M NaCl (pH 7.5). Each fraction was concentrated using Smart Evaporator C1 (BioChromato, Japan) and then desalted in PD Minitrap G25 (GE). Protein concentrations were measured by BCA method. Each fraction was subjected to SDS-PAGE on a 10%-20% gradient gel and stained with Coomassie Brilliant Blue. The band around 50-kD in purified fraction 13 was cut from the gel and identified by LC-MS/MS analysis.

### LC-MS/MS analysis

After destaining and washing of the excised gel pieces, proteins within the gel matrix were digested with trypsin at 37 °C for 20 h. Following digestion, the collected sample solution was subjected to LC-MS/MS analysis after desalting and concentration. Peptide separation was performed using Easy-nLC1200 liquid chromatograph (Thermo Fisher Scientific) with an analysis column (Thermo Fisher Scientific EASY-Spray column; 15 cm × 75 μm ID; 3 μm). The peptides were analyzed in a Q Exactive plus mass spectrometer (Thermo Fisher Scientific), eluted from the column with a linear solvent gradient (buffer A is 0.1% formic acid [FA]; buffer B is 80% acetonitrile/0.1% FA) for 20 min at a flow rate of 300 nl/min. The gradient profile was as follows: linear gradient from 5% to 35% buffer B over 10 min, 35% to 100% buffer B over 2 min, hold at 100% buffer B for 8 min. Protein identification was performed by searching the collected MS/MS data against a protein sequence database using a MASCOT Server. Search parameters included peptide mass tolerance (5 ppm), fragment mass tolerance (0.02 Da), and variable modifications (oxidation of methionine). The false discovery rate (FDR) thresholds for peptides were set to 1%.

## Analysis of amylolytic activity

The following two methods were used for α-amylase activity and starch degradation activity. α-amylase activity measured as a test tube method was performed using the α-amylase activity assay kit (Kikkoman, Japan). 2-Chloro-4-nitrophenyl 65-azido-65-deoxy-β-maltopentaoside used as a substrate was degraded by α-amylase to produce chloro-4-nitrophenyl β-maltosaccharides. 2-Chloro-4-nitrophenol produced from chloro-4-nitrophenyl β-maltosaccharides by the action of the conjugative enzymes, glucoamylase and β-glucosidase, was determined. Zymogram analysis was performed to detect α-amylase activity. In brief, SDS-PAGE, the gel was washed with H_2_O for 10 min. The gel was incubated twice with 1% starch solution at 60 °C for 1 h and cooled at 4 °C for 40 min. After washing with H_2_O, incubated with 0.15 M sodium citrate/phosphate buffer (pH4.8) for 2.5 h. Finally, the gel was stained with KI/I_2_ solution (10 g of KI, 1 g of I_2_ in 100 ml of H_2_O) for less than 5 min and washed with H_2_O. Transparent bands due to amylolytic activity appeared on the blue gel background. A negative control was prepared by heating the rice-*koji* extract solution at 100 °C for 10 min to inactivate enzymes. The complete loss of α-amylase activity after heat treatment was confirmed using the α-amylase activity assay kit. This heat-inactivated extract was then added to the culture medium under the same conditions as the untreated extract.

### Growth of *F. prausnitzii* in the modified FM supplemented with *A. oryzae*-derived α-amylase

To examine the comparative growth-promoting effect of rice-*koji* extract and purified α-amylase from *A. oryzae*, the OD590 and ATP production of *F. prausnitzii* were measured 15 h after inoculation in a modified FM containing 0.1% starch as the sole carbon source. The medium was supplemented with either 1% rice-*koji* extract, an equivalent amount (56.4 mU) of purified α-amylase (SIGMA), or H₂O (control). Starch concentration was set to 0.1%. A preliminary dose-dependency experiment confirmed that the growth-promoting effect of α-amylase at this starch concentration was not saturated with an amount of α-amylase equivalent to that in the 1% rice-*koji* extract (56.4 mU) (see Supplementary Fig. S2 online). This indicates that the 0.1% starch concentration is a sufficient amount for the 56.4 mU of α-amylase used in this study to exert its effect.

### Measurement of *n*-butyric acid in the culture

After 17 h of cultivation in the modified FM containing 0.1% starch as the sole carbon source, 2.5 ml each of the culture was centrifuged (15,000 × *g*, 5 min, 4 °C). The supernatant (2.0 ml) was collected and stored at -80 °C until use. *n*-Butyric acid in the culture supernatant were derivatized with 2-nitrophenylhydrazine using a YMC-Pack FA kit (YMC Co. Ltd., Japan) and were extracted with hexane and diethyl ether. Standards of butyrate were obtained from Fujifilm Wako Chemical Co Ltd. The high-performance liquid chromatography system consisted of a Waters e2695 separation module (0–29 MPa) and a Waters-UV detector connected in series was used. Chromatographic records were obtained using Gold Nouveau software, version 1.7 (Waters). Chromatographic separations were performed on YMC-FA-250 × 6 mm. Columns were purchased from YMC Co. Ltd. Injections (15 µl of sample) were performed using an autoinjector. Analysis was performed by continuous elution with acetonitrile: methanol: deionized water 30:16:54 (v/v/v) with the pH adjusted to 4.5 using 0.01 N HCl. The flow rate was constantly set to 0.5 ml/min. The monitored UV wavelength was 400 nm. Short chain fatty acids were monitored with UV detection.

### Statistical analysis

The data are expressed as the mean ± standard deviation. Differences between the control and other groups were evaluated by one-way analysis of variance (ANOVA) followed by Dunnett’s *post-hoc* test for multiple comparisons. For Fig. 5 and Supplementary Fig. S1B, comparisons among the groups were performed using a one-way ANOVA followed by Tukey’s *post-hoc* test for multiple comparisons. A *p*-value less than 0.05 was considered to indicate statistical significance.

## Supplementary Information

Below is the link to the electronic supplementary material.

Supplementary Material 1

## Data Availability

Data which have been used to create Figures can be provided upon reasonable request to the corresponding author.
