# Supplementary material for: α-Amylase in Aspergillus oryzae-fermented rice promotes the growth of human symbiotic Faecalibacterium Prausnitzii
Source: Sci Rep. 2026 Jan 20;16:5792. doi: 10.1038/s41598-026-36928-x (PMC12894947; doi:10.1038/s41598-026-36928-x)
Supplement: Supplementary file 1 — Supplementary Material 1 [file 41598_2026_36928_MOESM1_ESM.docx]

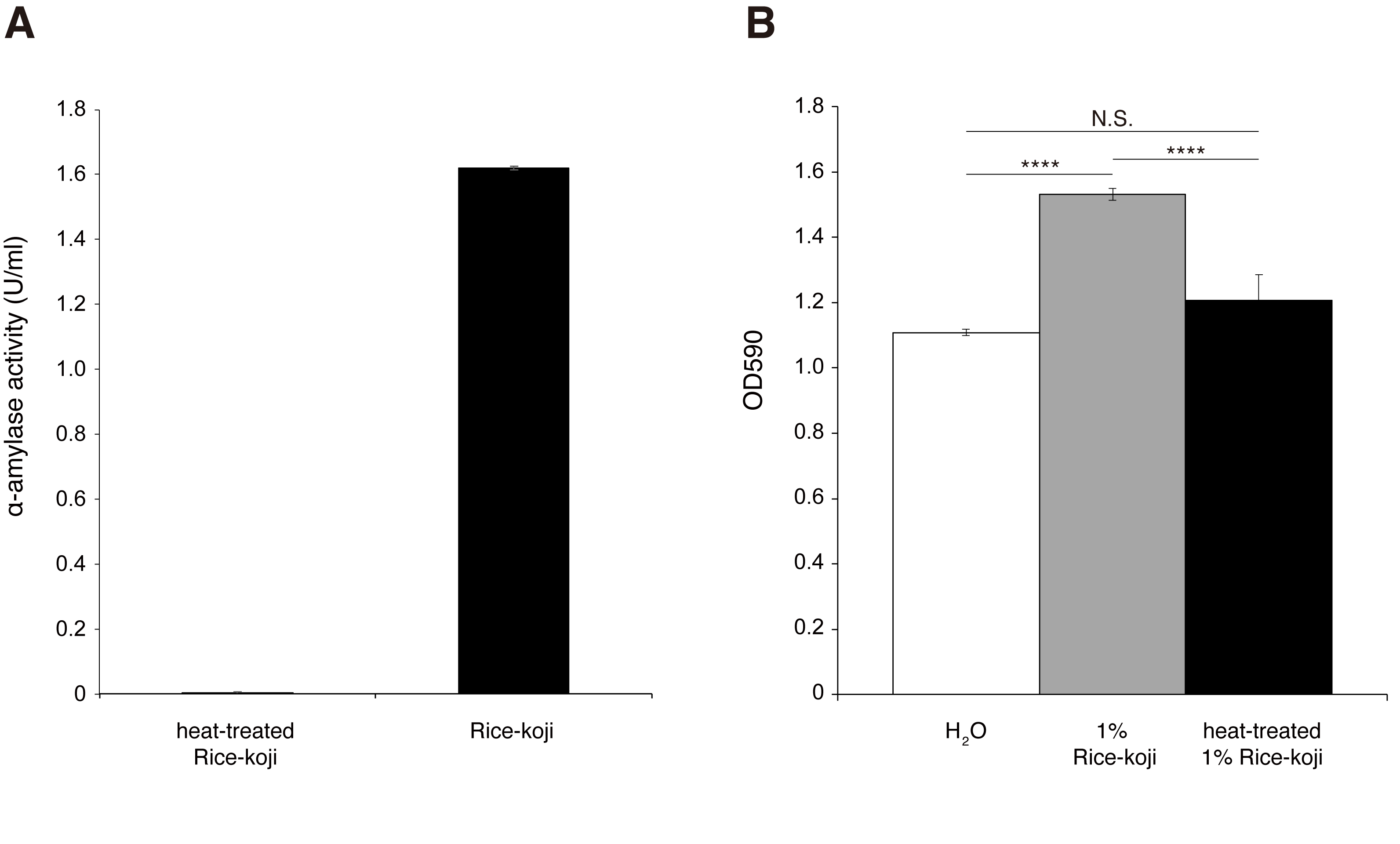


**Supplementary Fig. S1.** Effect of heat treatment on the α-amylase activity and growth-promoting effect of rice-*koji* extract. **(A)** α-amylase activity in the rice-*koji* extract before (Rice-*koji*) and after heat treatment at 100°C for 10 min (heat-treated Rice-*koji*). The activity was completely abolished by the heat treatment. **(B)** Growth of *F. prausnitzii* (OD590) after 19 h of cultivation in FM medium supplemented with H₂O (white), 1% rice-*koji* extract (light gray), or 1% heat-treated rice-*koji* extract (black). The growth-promoting effect was lost upon heat treatment. Statistical significance was determined by one-way ANOVA with Tukey's *post-hoc* test (****, *p* < 0.0001). N.S. indicates no significant difference.


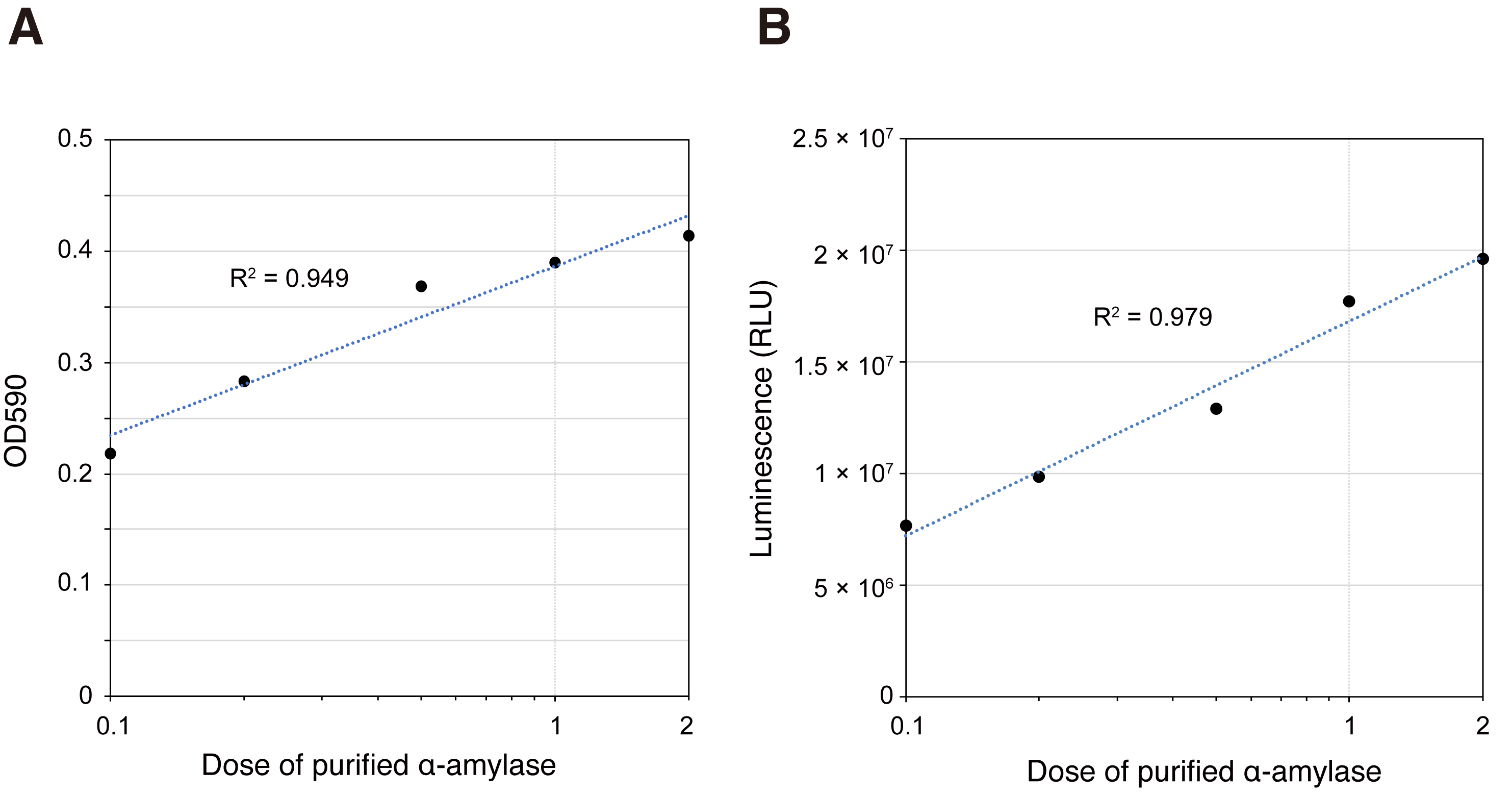


**Supplementary Fig. S2.** Dose-dependent growth-promoting effect of purified α-amylase on *F. prausnitzii*. *F. prausnitzii* was cultured for 15 h in a modified FM containing 0.1% starch as the sole carbon source, which was supplemented with varying doses of purified α-amylase from *A. oryzae*. The standard dose (1x) contained the enzyme activity equivalent to that in 1% rice-*koji* extract (56.4 mU). The *x*-axis represents the relative dose of α-amylase (range tested: 0.1x, 0.2x, 0.5x, 1x, and 2x), which are transformed into the enzyme a logarithmic scale. (A) Correlation between the supplemented dose of α-amylase and cell growth (OD590, R² = 0.949). (B) Correlation between the dose ofα-amylase added to the media and metabolic activity (ATP-dependent luminescence, RLU, R² = 0.979). Dotted lines represent the approximation correlation curves.


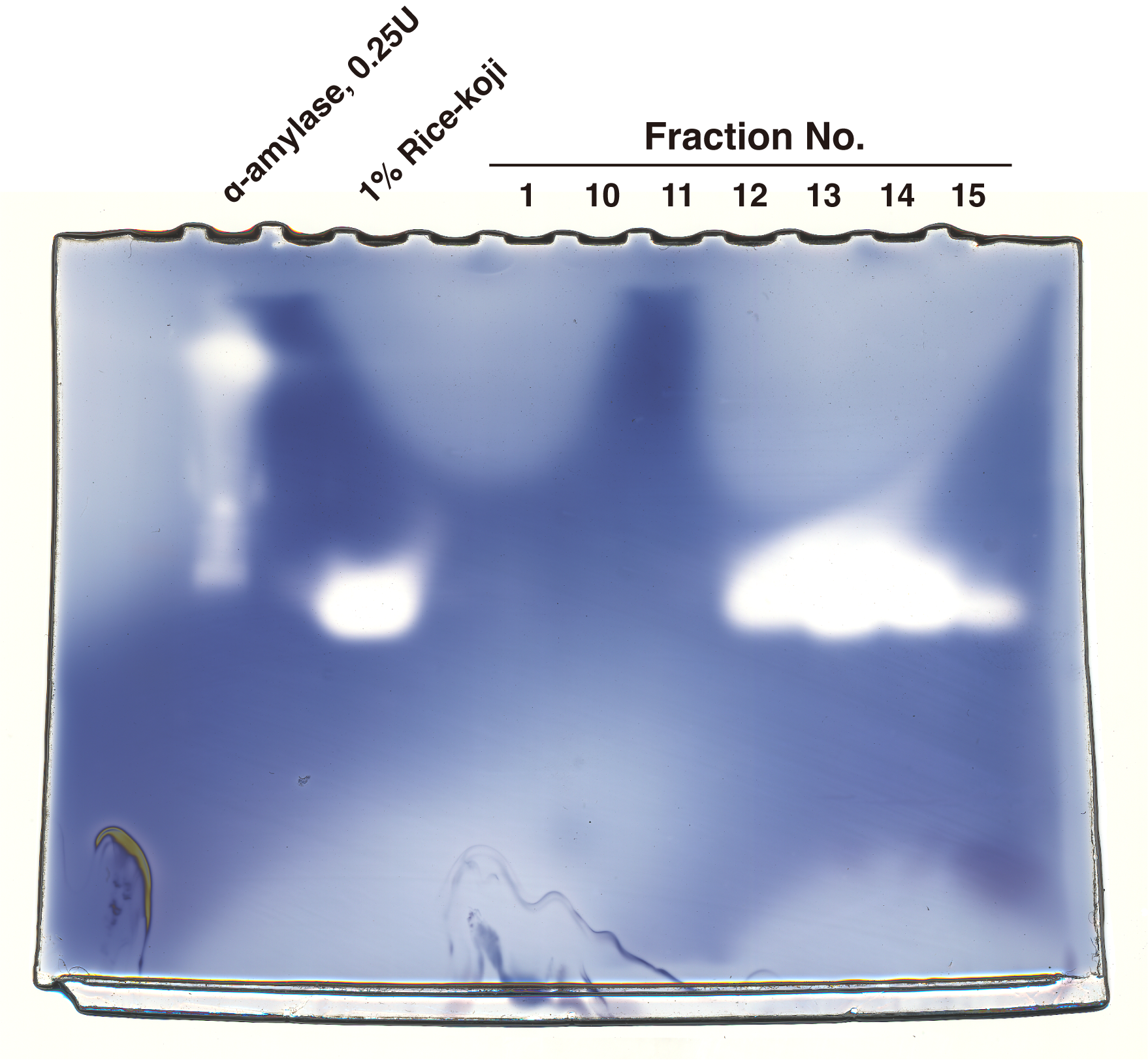


**Supplementary Fig. S3.** Uncropped full-length SDS-PAGE gels corresponding to **Fig. 3A**.
